# Acute appendicitis in the elderly: risk factors for perforation

**DOI:** 10.1186/1749-7922-9-6

**Published:** 2014-01-15

**Authors:** Abdelkarim H Omari, Muhammad R Khammash, Ghazi R Qasaimeh, Ahmad K Shammari, Mohammad K Bani Yaseen, Sahel K Hammori

**Affiliations:** 1 Department of General Surgery and Urology, King Abdullah University Hospital, Jordan University of Science and Technology, 22110 Irbid, Jordan; 2 Department of General Surgery, Princess Basma Teaching Hospital, 22110 Irbid, Jordan; 3 Department of General Surgery, Prince Rashid Military Teaching Hospital, 22110 Irbid, Jordan

**Keywords:** Acute appendicitis, Perforated appendix, Acute appendicitis in the elderly, Age and appendicitis, Peritonitis

## Abstract

**Background:**

Acute appendicitis is the most common surgical emergency and becomes serious when it perforates. Perforation is more frequent in the elderly patients. The aim of this study was to identify the risk factors of perforation in elderly patients who presented with acute appendicitis.

**Methodology:**

The medical records of 214 patients over the age of 60 years who had a pathologically confirmed diagnosis of acute appendicitis over a period of 10 years (2003-2013) were retrospectively reviewed. Patients were grouped into those with perforated and those with nonperforated appendicitis. Comparison was made between both groups in regard to demography, clinical presentation, and time delay to surgery, diagnosis, hospital stay and postoperative complications. Clinical assessment, Ultrasonography and Computerized tomography, in that order, were used for diagnosis. The incidence of perforation was also compared with a previous report from the same region 10 years earlier.

**Results:**

During the study period, a total of 214 patients over the age of 60 years had acute appendicitis, 103 males and 111 females. Appendix was found perforated in 87 (41%) patients, 46 (53%) males and 41 (47%) females. Of all patients, 31% were diagnosed by clinical assessment alone, 40% needed US and 29% CT scan. Of all the risk factors studied, the patient’s pre-hospital time delay was the most important risk factor for perforation. Perforation rate was not dependent on the presence of comorbid diseases or in-hospital time delay. Post operative complications occurred in 44 (21%) patients and they were three times more common in the perforated group, 33 (75%) patients in the perforated and 11 (25%) in the nonperforated group. There were 6 deaths (3%), 4 in the perforated and 2 in the nonperforated group.

**Conclusion:**

Acute appendicitis in elderly patients is a serious disease that requires early diagnosis and treatment. Appendiceal Perforation increases both mortality and morbidity. All elderly patients presented to the hospital with abdominal pain should be admitted and investigated. The early use of CT scan can cut short the way to the appropriate treatment.

## Introduction

Acute appendicitis is still the commonest abdominal surgical emergency with a lifetime incidence of 7%. Appendicitis is known to be the disease of the younger age groups with only 5-10% of cases occurring in the elderly population. However, the incidence of the disease in this age group seems to be rising due to recent increase in the life expectancy [1-11].

As compared to younger age group, elderly patients have more underlying diseases and sluggish bodily physiological reactions resulting in a higher rate of morbidity and mortality [1,2].

Furthermore, the often atypical presentation and delay in seeking medical help have been associated with delay in diagnosis and treatment resulting in high morbidity and mortality rates [3,4]. The prognosis of uncomplicated appendicitis in both young and old age groups is nearly equal. However, perforation worsens the condition dramatically resulting in higher rates of morbidity and mortality [5-8].

In order to improve our clinical understanding of the factors leading to perforation and to reduce its incidence if possible, we reviewed the medical records of all our patients over the age of 60 years with a pathologically confirmed acute appendicitis over the past 10 years. We determined the rate of appendiceal perforation and factors associated with perforation including demographic data, delayed presentation to medical care, delayed diagnosis and treatment and the presence of co-morbid diseases. Also, we studied the presenting symptoms and physical findings, laboratory investigation, use of radiological evaluation, complications and postoperative hospital stay.

A comparison was made between perforated and nonperforated groups regarding those variables. In addition, we compared our result with another study that was done in this region 10 years back.

## Methodology

The medical records of all patients (60 years and above) who underwent appendectomy at 3 major teaching hospitals in the north of Jordan from 1st January 2003 to the end of December 2012 were retrospectively reviewed. These three hospitals with a total of 1000 beds are affiliated to the Jordan University of Science and technology and draining an area of more than 1.5 million inhabitants. Data was collected through the computerized system of the King Abdulla University Hospital (KAUH) and manually from the patient’s registry of Princess Basma and Prince Rashid hospitals.

We identified all patients who underwent appendectomy over the above mentioned study period. On a case by case basis and with the help of the histopathological and operative reports, we excluded all patients who had normal or incidental appendectomies in addition to those with incomplete medical records.

Chart review was done to collect information on patient’s demographic data, initial clinical presentation and assessment, presence of co morbid diseases (diabetes mellitus, hypertension, cardiac, respiratory or renal diseases…etc), laboratory investigations, radiological studies with focus on Ultrasonography (US)and Computerized Tomography (CT) scan and whether the appendix was found perforated or not. Appendix was defined as perforated if it was described so in the operative notes and confirmed by the histopathological report.

At our three hospitals, patients with abdominal pain are usually seen first in the (ER) by the emergency physician and then by the surgeon on duty (if consulted) who admits or discharges the patient. Admitting diagnosis was based onhistory and clinical findings. These were defined as fever > 38°C, increased WBC > 10^9^/L and right lower abdominal pain. The decision to use additional imaging studies as US or CT scan is usually taken by the surgeon, results of which are interpreted by a certified radiologist. Diagnosis of acute appendicitis was made on the appearance of its wall, surrounding inflammation and edema with or without the presence of intra abdominal free fluid. CT scan study was usually spared for those cases when the Clinical Assessment (CA) and (US) were inconclusive. Once the diagnosis of acute appendicitis was made, the patient was given a shot of intravenous broad spectrum antibiotic that covers aerobic and anaerobic organisms and prepared for surgery. Open appendectomy was done for all patients, through Mc Burney’s or midline incisions. So far, neither the laparoscopic appendectomy nor the nonoperative management has been adopted for the treatment of acute appendicitis in the elderly patients at our hospitals.

The time interval from the onset of symptoms to the time of registration in the emergency room (ER) was coded in hours and defined as patient delay. The time from the (ER) visit to the operating room was defined as hospital delay and included time to diagnosis and time waiting for surgery.

Appendicitis was categorized into perforated (free or contained perforation, abscess formation) and nonperforated. A comparison between them was made in regard to demographic data, clinical presentation, investigations, patient’s delay, hospital delay and post operative hospital stay and complications. Also a comparison of the incidence of perforated appendicitis was made between our present study and another work that was done 10 years back in this region.

Computer program, Statistical Package for the Social Sciences (SPSS 16) was used for statistical analysis*. P*-*Value* < 0.05 was considered statistically significant when comparing variables.

Ethical approval was granted from the institution review board (IRB) of Jordan University of Science and Technology and King Abdullah University Hospital.

## Results

A total of 214 patients above the age of 60 years with histopathologically proven acute appendicitis during the period between January 2003 and December 2012 were analyzed retrospectively. There were 103 males and 111 females with a mean age of 64.4 ±2.7 years (range 60-95 years). A hundred and seventy seven (83%) patients were in their 60-69 years of age, 28 (13%) in the age group of 70-79, 8 (3%) patients in their 80-89 years and only one patient was 95 years old. Eighty seven (41%) patients proved to have perforated appendicitis, 46 (53%) males and 41 (47%) females (Table 1).

**Table 1:** Patient’s demographics, Co morbid diseases and post operative complications

**Characteristics**	**Total population**	**Perforated**	**Non-perforated**	**Post. op complication**
	**100%**	**41%**	**59%**	**21%**
Age	64.43 yr	65.23 yr	63.3 yr	64.3 yr
Sex				
Male	48	53	45)	61
Female	52	47	55	39
Co- morbidities	43	37	47	75
Diabetes	11	11	10	18
Hypertension	13	10	14	18
Cardiac diseases	12	9	16	18
Lung diseases	4	3	5	9
Renal diseases	2	2	2	7
Malignancies	1	2	1	5

Of all patients, there were 92 (43%) who had concurrent chronic medical diseases; Hypertension 27 (13%), chronic cardiac diseases 26 (12%), diabetes mellitus 23 (11%), chronic obstructive airway disease 9 (4%), end stage renal disease 4 (2%) and malignant diseases in 3 (1%) patients. No significant statistical differences between the risk of perforation and the presence of co morbid diseases were found (Table 1).

Regarding the time delay for treatment and as shown in Table 2, patients in the perforated group had a significantly longer Pre-hospital time delay than those in the nonperforated group (79.6 h and 47.3 h respectively) with <0.0001 *p*-value. At the same time, the table did not show a statistically significant difference between the two groups in regard to In-hospital delay (*p*-value 0.7923) (Table 2).

**Table 2:** Delay in surgical intervention and post operative mean hospital stay

**Variable**	**Perforated**	**Non perforated**	**P-value**
	***n*= (87)**	***n*= (127)**	
Mean delay in surgical treatment			
Pre hospital delay	79.6 ± 62.4 hr	47.3 ± 43.7 hr	< 0.0001*
Hospital delay	19.2 ± 10.3 hr	18.7 ± 15.5 hr	0.7923
Post op hosp stay	7.4 ± 6.3 days	4.2 ± 3.1 days	<0.0001*

*The result is significant.

Regarding the clinical presentation, all patients were complaining of abdominal pain. However, the typical migratory pain that starts around the umbilicus and shifts later to the right lower abdomen was described only by 101 (47%) patients, 75 (59%) patients in the nonperforated and 26 (30%) in the perforated group. Anorexia was present in 74% of all patients but it could not differentiate perforated from nonperforated groups. Nausea and vomiting were present in 57% of the patients and were more significantly found in the non perforated group (Table 3).

**Table 3:** Comparison between perforated and nonperforated groups in regard to clinical picture

**Variables**	**Total**	**Perforated**	**Non perforated**	**P-value**
	***n*=214 (100%)**	***n*= 87 (41%)**	***n*= 127 (59%)**	
**Migrating pain**	101 (47)	26 (30)	75 (59)	<0.0001*
**Anorexia**	150 (70)	64 (74)	86 (68)	0.3588
**Nausea & vomiting**	122 (57)	37 (43)	85 (67)	0.0004*
**Tender right lower abdomen**	180 (84)	65 (75)	115 (91)	0.0018*
**Rebound tenderness**	160 (75)	70 (80)	90 (71)	0.1125
**Fever > 38°C**	87 (41)	44 (51)	43 (34)	0.0145*
**WBC count**	143 (63)	62 (71)	72 (57)	0.0304*
**WBC shift to left**	159 (74)	82(94)	77 (61)	<0.0001*

*The result is significant.

Of all patients, 41% were febrile at presentation (>38°C). Fever was seen more in the perforated group of patients (51%-34%). Localized tenderness in the right lower abdomen was present in 84% of all patients with 91% in the nonperforated compared to 75% in the perforated group. Although rebound tenderness was found in 75% of patients, it did not differentiate between both groups (Table 3).

Increased WBC count > 10^9^/L, was seen in 143 (63%) of all patients at presentation. In the perforated group, Sixty two (71%) patients had high WBC with 94% shift to the left compared to 72 (57%) patients with 61% shift to the left in the non perforated group (Table 3).

Clinical Assessment (CA), Ultrasonography (US) and Computerized Tomography (CT) scan were used in that order for diagnosis. Of all patients 31% were diagnosed by CA alone, US detected another 40% and the remaining 29% were diagnosed by CT scan (Table 4). Although we couldn’t calculate the sensitivity and specificity of each diagnostic test as we studied the positive cases only, we found that there were no false positive results when CT scan was used.

**Table 4:** Number and percentage of patients diagnosed with appendicitis

**Variable**	**Total**	**Perforated**	**Nonperforated**
	***n*=214 (100%)**	***n*= 87 (41%)**	***n*= 127 (59%)**
Diagnostic tools:			
Clinical assessment	66 (31)	27 (31)	39 ( 31)
Ultrasonography	85 (40)	29 (33)	56 (44)
Computerized scan	63 (29)	31 (36)	32 (25)

Mc Burney’s incision was used in 168 and lower midline incision in 46 patients.

Post operative complications were seen in 44 (21%) patients. Complications were three times more frequent in the perforated as compared to the nonperforated group of patients, 33 (75%) and 11 (25%) respectively (Table 1). Four patients developed wound dehiscence and other eight had intra abdominal sepsis and collections, all in the perforated group except one. Other 22 patients in both groups had wound infection but all, except one, responded to antimicrobial treatment, debridement and dressings. Other complication as renal failure, chest infection, and respiratory failure, cardiovascular accidents were noted in both groups.

There were 6 (3%) deaths in both groups, four in the perforated and two in the nonperforated group. In the perforated group, two patients developed multiple intra abdominal abscess collections and died due to uncontrollable sepsis. Of the other two, one was already on chemotherapy treatment for lymphoma and died due to uncontrollable atypical pneumonia while the other had an advanced cardiovascular disease and died due to congestive heart failure. In the nonperforated group, one patient died due to uncontrolled intra abdominal sepsis and the other due to massive myocardial infarction. As expected, the hospital stay was longer for patients in the perforated group (7.4 ± 6.3 and 4.2 ±3.1 days in perforated and nonperforated groups respectively) (Table 2).

## Discussion

Acute appendicitis continues to be the commonest cause of surgical abdominal emergency. It was often thought to be the disease of the young but as a result of recent increases in lifetime expectancy, the incidence of acute appendicitis also increased in the elderly [1-11].

The incidence of appendiceal perforation in acute appendicitis is estimated to be in the range of 20-30% which increases to 32-72% in patients above 60 years of age [3-9,12-14]. The reasons behind this high rate were postulated to be due to the late and atypical presentation, delay in diagnosis and surgical intervention, presence of comorbid diseases and to the age-specific physiological changes [1-8,13,15-18]. In our study, perforated appendicitis was found in 87 (41%) patients, a result that lies within the range reported by many other reports [3,4,7,8,13,14,18]. Also found in the study was the absence of sex predilection for perforation; 46 (53%) patients were males and 41 (47%) were females. Although 92 (43%) of all patients had co morbid diseases at presentation, the risk of perforation did not appear to depend upon their presence (Table 1). These results were in conformity to the finding of Storm-Dickerson *et al.*[4].

Delay in presentation was found by many authors to be the reason behind the higher rate of perforation seen in the elderly population [2,3,6,7,13,15-17]. Our study showed that perforation rate correlated well with delayed presentation (pre-hospital delay) but did not correlate with the in-hospital delay.

The triad of right lower abdominal pain and tenderness, fever and leukocytosis is reported to be present in not more than 26% of patients above 60 years [4,19,20]. In this study, all patients presented to the hospital with abdominal pain. However, the classical migratory pain of appendicitis was present in only 47% of them. Localized tenderness in the right lower abdomen which is considered to be the most constant diagnostic physical sign for appendicitis was present in 84% of cases. Both features (migratory pain and localized tenderness) were seen more often in the nonperforated rather than in the perforated group (Table 3). This finding may be explained by the fact that patients with perforated appendix would show poor localization of pain as well as more generalized lower abdominal tenderness and guarding.

Our study showed that, fever (>38°C) was present in 41% of all patients and was much higher in the perforated group (Table 3), a result which is in agreement with the findings of other studies [4,6,21].

Also in the study, WBC was found elevated in 63% of all patients with 74% shifts to left. As expected, values were higher in the perforated group as 71% of them had high WBC with 94% shift to left (Table 3). Again, a result in agreement with many other studies [1,4,21].

There are many scoring systems that have been used in the diagnosis of acute appendicitis like Alvarado, Kharbanda and Lintula scores [22-24]. In general, these clinical scoring systems have better Likelihood ratios (LRs) than individual symptoms or signs alone. However, they don’t have sufficient discriminatory or predictive ability to routinely be used alone to diagnose appendicitis. They have been used to determine the need for further radiologic studies or as a guide for dictating clinical management [25-27]. The policy of our hospitals has not adopted the use of any scoring system so far.

Advances in diagnostic skills and improvements in diagnostic facilities (CT) scan and (US) advocated improving the diagnosis in patients with suspected appendicitis [16,20,28]. US can often diagnose an inflamed appendix and detects free fluid in the pelvis but this simple method is influenced by the operator’s experience, the body built and co-operation of the patient. The wider use of CT scan for patients with suspected appendicitis has been shown to improve the accuracy of the diagnosis and decrease the negative laparotomy rates [3,4,17]. Recent studies reported a high sensitivity of 91-99% in this age group [20]. Storm-Dickerson TL *et al.* reported that the incidence of perforation declined over the past 20 years from 72% to 51% in his patients due to the earlier use of CT scan [4]. In our patients, CT scan was only used in those with equivocal findings and in whom the diagnosis was not reached after repeated CA and US. We could not calculate the sensitivity and specificity of CA, US and CT scans in our patients because we studied the positive cases. However, we did not find any false positive result when the CT scan was used.

Elderly patients have a higher risk for both mortality and morbidity following appendectomy. It was estimated to be around 70% as compared to 1% in the general population [1,4,9-11].

In our study, the overall post operative complication rate was 21%, a figure which is a bit lower than 27-60% reported by others [6,20,29]. As expected, complications were three times more frequent in the perforated as compared to the nonperforated group. This finding is in consistency with several other studies that have shown that perforation per se was the most predictive factor for post operative morbidity in the elderly patients with acute appendicitis [1,7,14,20].

The mortality rate in elderly patients following perforated appendicitis was reported between 2.3%-10%. Death is often related to septic complications compounded by the patient’s co morbidities [3,6,7,29,30].

In this study, there were 6 (3%) deaths in both groups, four in the perforated and two in the nonperforated group. Three patients died due to septic complications while the others due to respiratory and cardiovascular causes.

As compared to younger age groups, the length of the hospital stay is usually longer in the elderly patients. This is usually ascribed to the higher rate of complications, prolonged need of antibiotics, treatment of other comorbidities and difficulties in communication [6,16,31]. Our result of 7.4 and 4.2 days for perforated and nonperforated groups was found in agreement with these studies.

When comparing our result to a previous study that was done in the same region 10 years back [32], we found that the incidence of appendiceal perforation did not decrease over the past ten years in spite of improved health care programs and diagnostic facilities. We think that this failure was due to the underestimation of the seriousness of the abdominal pain in this age group by both the patients and the primary health care providers.

Other factors that may influence the patient outcomes were not specifically addressed in this analysis, but are relevant to medical decision making in cases of appendicitis.

Reports in the literature had appeared describing the advantages of laparoscopic surgery over the open technique in terms of decreasing post operative pain, time to recovery, wound complications and post operative hospital stay, while others found that referring an elderly patient with complicated appendicitis to laparoscopic surgery will increase the operative time, conversion rate and length of hospital stay [19,31,33]. In a recent study published in 2013, Wray CJ *et al.* concluded that, the question of whether or not appendectomy should be performed via an open or laparoscopic technique has been inherently difficult to answer because both approaches offer similar advantages, namely, a small incision, low incidence of complications, a short hospital stay, and rapid return to normal activity [25]. At our hospitals, the laparoscopic approach has been adopted for the treatment of appendicitis in the younger age groups but so far, not for the elderly patients.

Despite the fact that appendectomy has been regarded as the standard treatment for appendicitis for more than 100 years, several reports have appeared in the literature over the last few years describing nonoperative management of acute, uncomplicated appendicitis. This conservative treatment which consists of nil by mouth, intravenous fluids and broad spectrum antibiotics had proved effective with less pain but had high recurrence rate, a risk that should be compared with the complications after appendectomy [27,34-38]. However, Wray CJ *et al.* considered that the available evidence regarding this nonoperative management is provocative and that level 1 data to suggest this is an alternative treatment option are not universally accepted [25]. Although the main object of our study was not the management of acute appendicitis in elderly patients, but after reviewing the literature, we think that the non operative management of acute appendicitis in this age group should be comprehensively studied.

The result of this study should be read with limitations. First, it is a retrospective study and in order to highlight the risk factors leading to appendiceal perforation one would ideally collect clinical data before and not after perforation occurred. Second, the rate of perforation differs according to the patient’s accessibility to medical health services.

## Conclusion

Acute appendicitis should still be considered in the differential diagnosis of abdominal pain in the elderly patients. Delay in presentation to the hospital is associated with higher rates of perforation and post operative complications. All elderly patients presented with abdominal pain should be admitted and investigated. The early use of CT scan can cut short the way to the appropriate treatment.

### Ethical approval

Institution Review Board (IRB) of the Jordan University of Science and Technology and King Abdullah University Hospital granted the approval for this work.

## Abbreviations

ER: Emergency room; CA: Clinical assessment; US: Ultrasonography; CT scan: Computed tomography scan.

## Competing interests

The authors declare that they have no competing interests.

## Authors’ contributions

AO: participated in collecting data, design and coordination of the study, helped to draft the manuscript and reviewed the literature. MK: participated in planning, design and coordination of the study. AS: participated in collecting data, GQ: participated in literature review and coordination, MB: collected data from princess Basma teaching Hospital, SH: collected data from Prince Rashid Military Hospital. All authors read and approved the final manuscript.

## References

[B1] Horattas M Guyton D Diane W A reappraisal of appendicitis in theelderly Am J Surg 1990 160 291 293 10.1016/S0002-9610(06)80026-7 2393058

[B2] Smithy WB Wexner SD Daily TH The diagnosis and treatment of acute appendicitis in the aged Dis Colon Rectum 1986 29 170 173 10.1007/BF02555015 3943430

[B3] Franz MG Norman J Fabri PJ Increased morbidity of appendicitis with advancing age Am Surg 1995 61 40 44 7832380

[B4] Storm-Dickerson TL Horattas MC What we have learned over the past 20 years about appendicitis in the elderly? Am J Surg 2003 185 198 201 10.1016/S0002-9610(02)01390-9 12620555

[B5] Lunca S Bouras G Romedea NS Acute appendicitis in the elderly patient: diagnostic problems, prognostic factors and out-comes Rom J Gastroenterol 2004 13 299 303 15624027

[B6] Lee JF Leow CK Lau WY Appendicitis in the elderly ANZ J Surg 2000 70 593 596 10.1046/j.1440-1622.2000.01905.x 10945554

[B7] Sherlock DJ Acute appendicitis in the over-sixty age group Br J Surg 1985 72 245 246 10.1002/bjs.1800720337 3842605

[B8] Lau WY Fan ST Yiu TF Chu KW Lee JM Acute appendicitis in the elderly SurgGynecolObstet 1985 161 157 160 4023896

[B9] Yamini D Vargas H Bongard F Klein S Stamos MJ Perforated appendicitis: is ittruly a surgical urgency? Am Surg 1998 64 970 975 9764704

[B10] Hardin D Acute appendicitis: review and update Am FamPhys 1999 60 2027 2036 10569505

[B11] Tehrani H Petros JG Kumar RR Chu Q Markers of severe appendicitis Am Surg 1999 65 453 455 10231216

[B12] Temple C Huchcroft S Temple W The natural history of appendicitisin adults, a prospective study Ann Surg 1995 221 279 282 10.1097/00000658-199503000-00010 PMC1234570 7717781

[B13] Ryden CI Grunditz T Janzon L Acute appendicitis in patients above and below 60 years of age Acta ChirScand 1983 149 165 170 6880551

[B14] Paajanen H Kettunen J Kostiainen S Emergency appendictomies in patients over 80 years Am Surg 1994 60 950 953 7992972

[B15] Watters JM Blackslee JM March RJ Redmond ML The influence of age on the severity of peritonitis Can J Surg 1996 39 142 146 8769925 PMC3949853

[B16] Korner H Sondenaa K Soreide JA Andersen E Nysted A Lende TH Kiellevold KH Incidence of acute nonperforated and perforated appendicitis: age-specific and sex-specific analysis World J Surg 1997 21 313 317 10.1007/s002689900235 9015177

[B17] Eldar S Nash E Sabo E Matter I Kunin J Mogilner JG Abrahamson J Delay of surgery in acute appendicitis Am J S 1997 173 194 198 10.1016/s0002-9610(96)00011-6 9124625

[B18] Thorbjarnarson B Loehr WJ Acute appendicitis in patients over the age of sixty SurgGynecolObstet 1967 125 1277 1280 6065264

[B19] Paranjape C Dalia S Pan J Horattas M Appendicitis in the elderly: a change in the laparoscopic era SurgEndosc 2007 21 777 781 10.1007/s00464-006-9097-4 17285390

[B20] Pooler BD Lawrence EM Pickhardt PJ MDCT for suspected appendicitis in the elderly: diagnostic performance and patient outcome Emerg Radio 2012 19 27 33 10.1007/s10140-011-1002-3 22131057

[B21] Sheu BF Chiu TF Chen JC Tung MS Chang MW Young YR Risk factors associated with perforated appendicitis in elderly patients presenting with signs and symptoms of acute appendicitis ANZ J Surg 2007 77 662 666 10.1111/j.1445-2197.2007.04182.x 17635280

[B22] Alvarado A A practical score for the early diagnosis of acute appendicitis Ann Emerg Med 1986 15 557 564 10.1016/S0196-0644(86)80993-3 3963537

[B23] Kharabanda AB Taylor GA Fishman SJ Bachur RG A clinical decision rule to identify children at low risk of appendicitis Pediatrics 2005 116 709 716 10.1542/peds.2005-0094 16140712

[B24] Lintula H Kokki H Pulkkinen J Kettunen R Grohn O Eskelinen M Diagnostic score in acute appendicitis. Validation of a diagnostic score (Lintula score) for adults with suspected appendicitis Langenbecks Arch surg 2010 395 495 500 10.1007/s00423-010-0627-0 20379739

[B25] Wray CJ Kao LS Millas SG Tsao K Ko TC Acute appendicitis: controversies in diagnosis and management CurrProblSurg 2013 50 54 86 10.1067/j.cpsurg.2012.10.001 23374326

[B26] Rezak A Abbas HM Ajemian MS Dudrick SJ Kwasnik EM Decreased use of computed tomography with a modified clinical scoring system in diagnosis of pediatric acute appendicitis Arch Surg 2011 146 64 67 10.1001/archsurg.2010.297 21242447

[B27] Farahnak M Talaei-Khoei M Gorouhi F Jalali A The Alvarado score and antibiotics therapy as a corporate protocol versus conventional clinical management: randomized controlled pilot study of approach to acute appendicitis Am J Emerg Med 2007 25 850 852 10.1016/j.ajem.2007.01.012 17870498

[B28] Ilves I Paajanen HE Herzig KH Fagerstrom A Miettinen PJ Changing incidence of acute appendicitis and nonspecific abdominal pain between 1987 and 2007 in Finland World J Surg 2011 35 731 738 10.1007/s00268-011-0988-8 21327601

[B29] Freund HR Rubinstein E Appendicitis in the aged: is it really different? Am Surg 1984 50 573 576 6486575

[B30] Blomqvist PG Andersson RE Granath F Lambe MP Ekbom AR Mortality after appendectomy in Sweden, 1987-1996 Ann Surg 2001 233 455 460 10.1097/00000658-200104000-00001 11303128 PMC1421275

[B31] Kirstein B Perry ZH Mizrahi S Lantsberg L Value of laparoscopic appendectomy in the elderly patient World J Surg 2009 5 918 922 10.1007/s00268-008-9916-y 19172345

[B32] Qasaimeh GR Khader Y Matalqah I Nimri S Acute appendicitis in north of Jordan- A 10 year survey J Med J 2004 42 149 154

[B33] Hui TT Major KM Avital I Hiatt JR Margulies DR Outcome of elderly patients with appendicitis- effect of computed tomography and laparoscopy Arch Surg 2002 137 995 998 12215147 10.1001/archsurg.137.9.995

[B34] Hansson J Korner U Khorram-Manesh A Solberg A Lundholm K Randomized clinical trial of antibiotic therapy versus appendicectomy as primary treatment of acute appendicitis in unselected patients Br J Surg 2009 96 473 481 10.1002/bjs.6482 19358184

[B35] Malik AA Bari SU Conservative management of acute appendicitis J GastrointestSurg 2009 13 966 970 10.1007/s11605-009-0835-5 19277796

[B36] Styrud J Eriksson S Nilsson I Ahlberg G Haapaniemi S Neovius G Rex L Badume I Granstrom L Appendectomy versus antibiotic treatment in acute appendicitis. a prospective multicenter randomized controlled trial World J Surg 2006 30 1033 1037 10.1007/s00268-005-0304-6 16736333

[B37] Papandria D Goldstein SD Rhee D Salazar JH Arlikar J Gorgy A Ogtega G Zhang Y Abdullah F Risk of perforation increases with delay in recognition and surgery for acute appendicitis J Surg Res 2013 184 723 729 10.1016/j.jss.2012.12.008 23290595 PMC4398569

[B38] Liu K Fogg L Use of antibiotics alone for treatment of uncomplicated acute appendicitis: a systemic review and meta-analysis Surgery 2011 150 673 683 10.1016/j.surg.2011.08.018 22000179

